# 3-(2-Methyl-2-nitro­prop­yl)-1*H*-indole

**DOI:** 10.1107/S1600536812020582

**Published:** 2012-05-16

**Authors:** Zheng Fang, Feng Zhang, Bao-hua Zou, Kai Guo

**Affiliations:** a School of Pharmaceutical Sciences, Nanjing University of Technology, Puzhu South Road No. 30 Nanjing, Nanjing 210009, People’s Republic of China; b College of Life Science and Pharmaceutical Engineering, Nanjing University of Technology, Puzhu South Road No. 30 Nanjing, Nanjing 210009, People’s Republic of China

## Abstract

In the title compound, C_12_H_14_N_2_O_2_, the indole ring is essentially planar, with an r.m.s. deviation of 0.0136 Å. In the crystal, pairs of N—H⋯O hydrogen bonds link the mol­ecules into inversion dimers..

## Related literature

The title compound is an inter­mediate of the β-adrenergic receptor antagonist (β blocker) bucindolol {systematic name: 1-[[2-(3-indol­yl)-1,1-dimethyl­eth­yl]amino]-3-(2-nitrilear­yloxy)-2-propanol)}, see: Qiu *et al.*, (2003 ▶). For synthetic procedures, see: Kerighbaum *et al.* (1980 ▶). For a related structure, see: Léger *et al.* (1984 ▶).
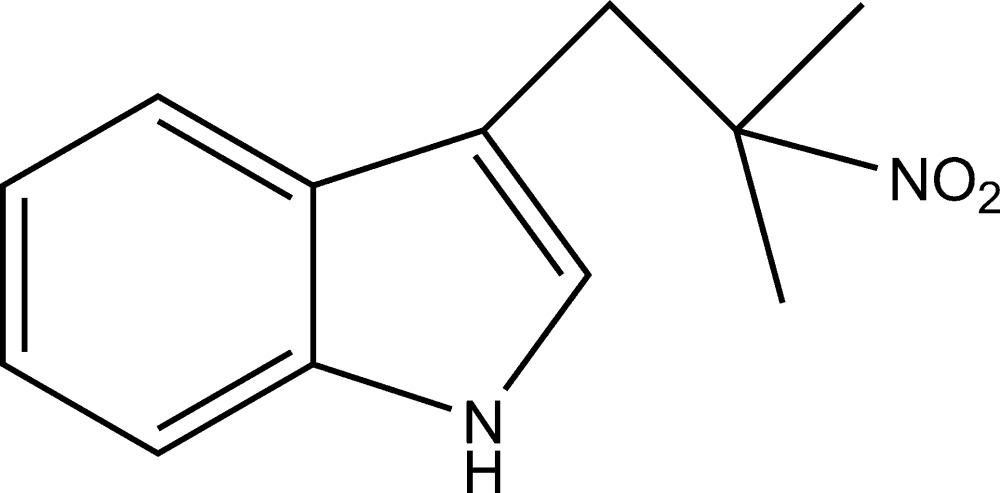


## Experimental

### 

#### Crystal data

C_12_H_14_N_2_O_2_
*M* *_r_* = 218.25
Monoclinic, 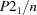

*a* = 6.1170 (12) Å
*b* = 10.123 (2) Å
*c* = 18.868 (4) Å
β = 91.36 (3)°
*V* = 1168.0 (4) Å^3^
*Z* = 4
Mo *K*α radiation
μ = 0.09 mm^−1^
*T* = 293 K
0.20 × 0.20 × 0.10 mm

#### Data collection

Enraf–Nonius CAD-4 diffractometer
Absorption correction: ψ scan (North *et al.*, 1968 ▶) *T*_min_ = 0.983, *T*_max_ = 0.992
2354 measured reflections
2141 independent reflections
1089 reflections with *I* > 2σ(*I*)
*R*_int_ = 0.029
3 standard reflections every 200 reflections intensity decay: 1%

#### Refinement

*R*[*F* ^2^ > 2σ(*F* ^2^)] = 0.057
*wR*(*F* ^2^) = 0.179
*S* = 1.00
2141 reflections
146 parameters
H-atom parameters constrained
Δρ_max_ = 0.14 e Å^−3^
Δρ_min_ = −0.13 e Å^−3^

### 

Data collection: *CAD-4 Software* (Enraf–Nonius, 1989 ▶); cell refinement: *CAD-4 Software*; data reduction: *XCAD4* (Harms & Wocadlo, 1995 ▶); program(s) used to solve structure: *SHELXS97* (Sheldrick, 2008 ▶); program(s) used to refine structure: *SHELXL97* (Sheldrick, 2008 ▶); molecular graphics: *SHELXTL* (Sheldrick, 2008 ▶); software used to prepare material for publication: *PLATON* (Spek, 2009 ▶).

## Supplementary Material

Crystal structure: contains datablock(s) global, I. DOI: 10.1107/S1600536812020582/pv2541sup1.cif

Structure factors: contains datablock(s) I. DOI: 10.1107/S1600536812020582/pv2541Isup2.hkl

Supplementary material file. DOI: 10.1107/S1600536812020582/pv2541Isup3.cml

Additional supplementary materials:  crystallographic information; 3D view; checkCIF report

## Figures and Tables

**Table 1:** Hydrogen-bond geometry (Å, °)

*D*—H⋯*A*	*D*—H	H⋯*A*	*D*⋯*A*	*D*—H⋯*A*
N1—H1*A*⋯O2^i^	0.86	2.55	3.187 (4)	132

Symmetry code: (i) 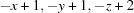.

**Fig. 1.**
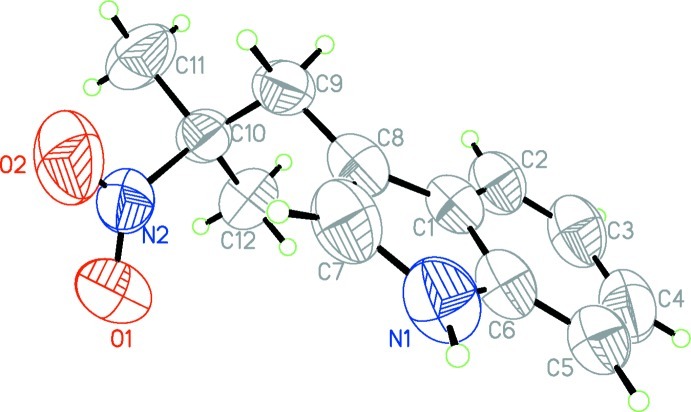
The molecular structure of the title compound with the atom numbering scheme. Displacement ellipsoids are drawn at the 50% probability level. H atoms are presented as small spheres of arbitrary radius.

**Fig. 2.**
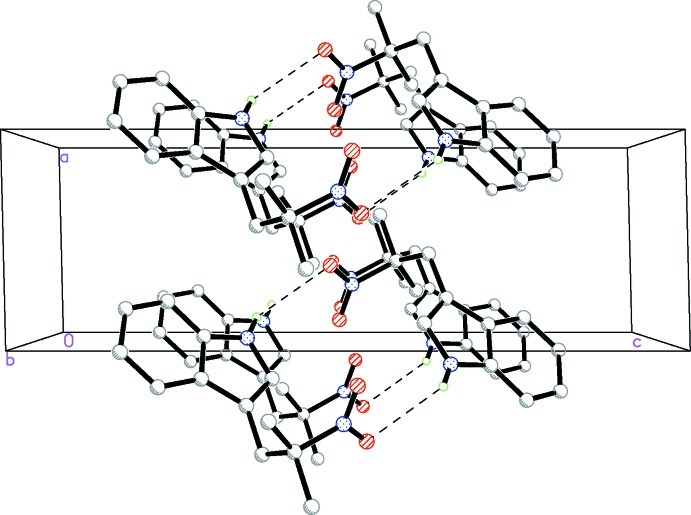
A view of the N—H···O hydrogen bonds (dotted lines) in the crystal structure of the title compound. H atoms non-participating in hydrogen- bonding were omitted for clarity.

## supplementary crystallographic information

### Comment

Bucindolol, 1-[[2-(3-indolyl)-1,l-dimethylethyl]-amino]-3-(2-nitrile-aryloxy)-2-propanol, is one of the β-adrenergic receptor antagonists (β blocker) for the treatment of essential hypertension (Qiu *et al.*, 2003). As a part of our studies on the synthesis of Bucindolol, the title compound (Fig. 1) which is used as the key intermediate, has been synthesized and its crystal structure reported in this article. The crystal structure of the title compound is stabilized by N1—H1A···O2 hydrogen bonds resulting in dimers of molecules lying about inversion centers (Fig. 1 and Tab. 1). The bond distances and angles in the title molecule are in excellent agreement with the corresponding bond distances and angles reported for a related structure (Léger *et al.*, 1984).

### Experimental

A mixture of gramine (13.0 g,(0.069 mol), 2-nitropropane (44 g,(0.49 mol), and NaOH pellets (2.9 g, 0.072 mol) was stirred and heated at reflux for 18 h. After the mixture had cooled to 298 K, 10% HOAc (60 ml) was added and stirring was continued for 1 h. The mixture was partitioned between 150 ml each of EtOH and water to afford an organic layer, which was separated, washed three times with water, and dried over MgS0_4_. Evaporation afforded 16.5 g of dark oil which slowly crystallized on standing at 298 K. Recrystallization of the crude product from EtOH-H_2_0 (1:1) gave 12.6 g (78%) of the title compound as pure yellow crystals (Kerighbaum *et al.*, 1980). Crystals of the title compound suitable for X-ray diffraction were obtained by slow evaporation of an ethanol solution.

### Refinement

All H atoms were positioned geometrically and refined using a riding model, with N—H = 0.86 Å and C—H = 0.93, 0.96 and 0.97 Å, for aryl, methyl and methylene H-atoms, respectively. The *U*_iso_(H) were allowed at 1.5*U*_eq_(methyl C) or 1.2*U*_eq_(non-methyl C/N).

### Crystal data

**Table:**

C_12_H_14_N_2_O_2_	*F*(000) = 464
*M**_r_* = 218.25	*D*_x_ = 1.241 Mg m^−^^3^
Monoclinic, *P*2_1_/*n*	Mo *K*α radiation, λ = 0.71073 Å
Hall symbol: -P 2yn	Cell parameters from 25 reflections
*a* = 6.1170 (12) Å	θ = 9–13°
*b* = 10.123 (2) Å	µ = 0.09 mm^−^^1^
*c* = 18.868 (4) Å	*T* = 293 K
β = 91.36 (3)°	Block, yellow
*V* = 1168.0 (4) Å^3^	0.20 × 0.20 × 0.10 mm
*Z* = 4	

### Data collection

**Table:**

Enraf–Nonius CAD-4 diffractometer	1089 reflections with *I* > 2σ(*I*)
Radiation source: fine-focus sealed tube	*R*_int_ = 0.029
Graphite monochromator	θ_max_ = 25.4°, θ_min_ = 2.2°
ω/2θ scans	*h* = 0→7
Absorption correction: ψ scan (North *et al.*, 1968)	*k* = 0→12
*T*_min_ = 0.983, *T*_max_ = 0.992	*l* = −22→22
2354 measured reflections	3 standard reflections every 200 reflections
2141 independent reflections	intensity decay: 1%

### Refinement

**Table:**

Refinement on *F*^2^	Secondary atom site location: difference Fourier map
Least-squares matrix: full	Hydrogen site location: inferred from neighbouring sites
*R*[*F*^2^ > 2σ(*F*^2^)] = 0.057	H-atom parameters constrained
*wR*(*F*^2^) = 0.179	*w* = 1/[σ^2^(*F*_o_^2^) + (0.084*P*)^2^] where *P* = (*F*_o_^2^ + 2*F*_c_^2^)/3
*S* = 1.00	(Δ/σ)_max_ < 0.001
2141 reflections	Δρ_max_ = 0.14 e Å^−^^3^
146 parameters	Δρ_min_ = −0.13 e Å^−^^3^
0 restraints	Extinction correction: *SHELXL97* (Sheldrick, 2008), Fc^*^=kFc[1+0.001xFc^2^λ^3^/sin(2θ)]^-1/4^
Primary atom site location: structure-invariant direct methods	Extinction coefficient: 0.043 (7)

### Special details

**Table:**

Geometry. All e.s.d.'s (except the e.s.d. in the dihedral angle between two l.s. planes) are estimated using the full covariance matrix. The cell e.s.d.'s are taken into account individually in the estimation of e.s.d.'s in distances, angles and torsion angles; correlations between e.s.d.'s in cell parameters are only used when they are defined by crystal symmetry. An approximate (isotropic) treatment of cell e.s.d.'s is used for estimating e.s.d.'s involving l.s. planes.
Refinement. Refinement of *F*^2^ against ALL reflections. The weighted *R*-factor *wR* and goodness of fit *S* are based on *F*^2^, conventional *R*-factors *R* are based on *F*, with *F* set to zero for negative *F*^2^. The threshold expression of *F*^2^ > σ(*F*^2^) is used only for calculating *R*-factors(gt) *etc*. and is not relevant to the choice of reflections for refinement. *R*-factors based on *F*^2^ are statistically about twice as large as those based on *F*, and *R*- factors based on ALL data will be even larger.

### Fractional atomic coordinates and isotropic or equivalent isotropic displacement parameters (Å^2^)

**Table:**

	*x*	*y*	*z*	*U*_iso_*/*U*_eq_	
O1	0.3793 (4)	0.2257 (3)	1.01215 (14)	0.1125 (10)	
C1	0.3885 (5)	0.3891 (3)	0.78945 (14)	0.0565 (8)	
N1	0.5544 (5)	0.5489 (3)	0.85178 (14)	0.0795 (9)	
H1A	0.6445	0.6098	0.8650	0.095*	
O2	0.1178 (5)	0.3613 (3)	1.02354 (13)	0.0997 (9)	
C2	0.3631 (5)	0.3039 (3)	0.73199 (16)	0.0680 (9)	
H2A	0.2434	0.2474	0.7288	0.082*	
N2	0.2086 (5)	0.2746 (3)	0.99150 (14)	0.0704 (8)	
C3	0.5157 (7)	0.3042 (4)	0.68028 (18)	0.0818 (11)	
H3A	0.4991	0.2472	0.6419	0.098*	
C4	0.6966 (7)	0.3888 (4)	0.68414 (19)	0.0860 (11)	
H4A	0.7994	0.3858	0.6487	0.103*	
C5	0.7254 (6)	0.4763 (4)	0.73929 (19)	0.0784 (10)	
H5A	0.8442	0.5335	0.7418	0.094*	
C6	0.5676 (5)	0.4748 (3)	0.79116 (16)	0.0651 (8)	
C7	0.3738 (6)	0.5096 (3)	0.88767 (17)	0.0742 (10)	
H7A	0.3307	0.5450	0.9306	0.089*	
C8	0.2668 (5)	0.4125 (3)	0.85229 (14)	0.0593 (8)	
C9	0.0639 (5)	0.3434 (3)	0.87513 (15)	0.0681 (9)	
H9A	−0.0179	0.3157	0.8330	0.082*	
H9B	−0.0259	0.4065	0.9000	0.082*	
C10	0.0990 (4)	0.2237 (3)	0.92255 (15)	0.0594 (8)	
C11	−0.1206 (5)	0.1680 (4)	0.9453 (2)	0.0956 (13)	
H11A	−0.2075	0.2377	0.9646	0.143*	
H11B	−0.0964	0.1010	0.9806	0.143*	
H11C	−0.1961	0.1303	0.9049	0.143*	
C12	0.2438 (5)	0.1190 (3)	0.89062 (18)	0.0751 (10)	
H12A	0.3789	0.1583	0.8767	0.113*	
H12B	0.1709	0.0811	0.8498	0.113*	
H12C	0.2732	0.0511	0.9251	0.113*	

### Atomic displacement parameters (Å^2^)

**Table:**

	*U*^11^	*U*^22^	*U*^33^	*U*^12^	*U*^13^	*U*^23^
O1	0.0832 (17)	0.165 (3)	0.0884 (18)	0.013 (2)	−0.0211 (15)	−0.0015 (18)
C1	0.0663 (19)	0.0544 (17)	0.0482 (17)	0.0097 (16)	−0.0089 (14)	−0.0007 (14)
N1	0.103 (2)	0.0663 (18)	0.0686 (18)	−0.0168 (17)	−0.0142 (16)	−0.0007 (15)
O2	0.131 (2)	0.104 (2)	0.0646 (15)	0.0075 (17)	0.0064 (14)	−0.0244 (15)
C2	0.085 (2)	0.065 (2)	0.0542 (18)	0.0045 (18)	−0.0038 (16)	−0.0053 (16)
N2	0.0663 (18)	0.087 (2)	0.0576 (16)	−0.0094 (17)	0.0052 (14)	0.0047 (16)
C3	0.115 (3)	0.073 (2)	0.0575 (19)	0.018 (2)	0.005 (2)	−0.0036 (18)
C4	0.099 (3)	0.094 (3)	0.066 (2)	0.024 (3)	0.015 (2)	0.019 (2)
C5	0.080 (2)	0.079 (2)	0.076 (2)	0.001 (2)	−0.007 (2)	0.024 (2)
C6	0.079 (2)	0.0619 (19)	0.0543 (18)	0.0063 (19)	−0.0058 (17)	0.0084 (16)
C7	0.099 (3)	0.069 (2)	0.0540 (19)	0.004 (2)	−0.0044 (19)	−0.0060 (17)
C8	0.072 (2)	0.0553 (18)	0.0500 (17)	0.0110 (17)	−0.0094 (15)	−0.0021 (15)
C9	0.0590 (19)	0.091 (2)	0.0543 (18)	0.0182 (18)	−0.0052 (14)	−0.0103 (17)
C10	0.0523 (17)	0.071 (2)	0.0552 (17)	−0.0026 (16)	−0.0003 (14)	−0.0109 (15)
C11	0.062 (2)	0.117 (3)	0.108 (3)	−0.020 (2)	0.020 (2)	−0.020 (3)
C12	0.075 (2)	0.068 (2)	0.083 (2)	−0.0041 (18)	0.0151 (18)	−0.0052 (18)

### Geometric parameters (Å, º)

**Table:**

O1—N2	1.212 (3)	C5—H5A	0.9300
C1—C2	1.391 (4)	C7—C8	1.349 (4)
C1—C6	1.397 (4)	C7—H7A	0.9300
C1—C8	1.435 (4)	C8—C9	1.497 (4)
N1—C7	1.369 (4)	C9—C10	1.519 (4)
N1—C6	1.372 (4)	C9—H9A	0.9700
N1—H1A	0.8600	C9—H9B	0.9700
O2—N2	1.208 (3)	C10—C12	1.516 (4)
C2—C3	1.366 (4)	C10—C11	1.528 (4)
C2—H2A	0.9300	C11—H11A	0.9600
N2—C10	1.538 (4)	C11—H11B	0.9600
C3—C4	1.400 (5)	C11—H11C	0.9600
C3—H3A	0.9300	C12—H12A	0.9600
C4—C5	1.374 (5)	C12—H12B	0.9600
C4—H4A	0.9300	C12—H12C	0.9600
C5—C6	1.391 (5)		
			
C2—C1—C6	118.4 (3)	C7—C8—C1	105.9 (3)
C2—C1—C8	134.1 (3)	C7—C8—C9	126.4 (3)
C6—C1—C8	107.5 (3)	C1—C8—C9	127.7 (3)
C7—N1—C6	108.5 (3)	C8—C9—C10	115.8 (2)
C7—N1—H1A	125.7	C8—C9—H9A	108.3
C6—N1—H1A	125.7	C10—C9—H9A	108.3
C3—C2—C1	119.3 (3)	C8—C9—H9B	108.3
C3—C2—H2A	120.3	C10—C9—H9B	108.3
C1—C2—H2A	120.3	H9A—C9—H9B	107.4
O2—N2—O1	122.6 (3)	C12—C10—C9	113.6 (2)
O2—N2—C10	118.0 (3)	C12—C10—C11	112.3 (3)
O1—N2—C10	119.3 (3)	C9—C10—C11	110.3 (3)
C2—C3—C4	121.1 (3)	C12—C10—N2	108.9 (2)
C2—C3—H3A	119.4	C9—C10—N2	106.5 (2)
C4—C3—H3A	119.4	C11—C10—N2	104.8 (2)
C5—C4—C3	121.4 (3)	C10—C11—H11A	109.5
C5—C4—H4A	119.3	C10—C11—H11B	109.5
C3—C4—H4A	119.3	H11A—C11—H11B	109.5
C4—C5—C6	116.6 (4)	C10—C11—H11C	109.5
C4—C5—H5A	121.7	H11A—C11—H11C	109.5
C6—C5—H5A	121.7	H11B—C11—H11C	109.5
N1—C6—C5	129.5 (3)	C10—C12—H12A	109.5
N1—C6—C1	107.3 (3)	C10—C12—H12B	109.5
C5—C6—C1	123.2 (3)	H12A—C12—H12B	109.5
C8—C7—N1	110.8 (3)	C10—C12—H12C	109.5
C8—C7—H7A	124.6	H12A—C12—H12C	109.5
N1—C7—H7A	124.6	H12B—C12—H12C	109.5
			
C6—C1—C2—C3	1.7 (4)	C2—C1—C8—C7	−179.3 (3)
C8—C1—C2—C3	−178.4 (3)	C6—C1—C8—C7	0.6 (3)
C1—C2—C3—C4	−0.1 (5)	C2—C1—C8—C9	1.2 (5)
C2—C3—C4—C5	−1.2 (5)	C6—C1—C8—C9	−178.8 (3)
C3—C4—C5—C6	0.9 (5)	C7—C8—C9—C10	−87.7 (4)
C7—N1—C6—C5	−178.0 (3)	C1—C8—C9—C10	91.6 (3)
C7—N1—C6—C1	0.8 (3)	C8—C9—C10—C12	−56.2 (3)
C4—C5—C6—N1	179.4 (3)	C8—C9—C10—C11	176.7 (3)
C4—C5—C6—C1	0.8 (4)	C8—C9—C10—N2	63.6 (3)
C2—C1—C6—N1	179.1 (3)	O2—N2—C10—C12	178.4 (3)
C8—C1—C6—N1	−0.9 (3)	O1—N2—C10—C12	−2.7 (4)
C2—C1—C6—C5	−2.0 (4)	O2—N2—C10—C9	55.6 (3)
C8—C1—C6—C5	178.0 (3)	O1—N2—C10—C9	−125.5 (3)
C6—N1—C7—C8	−0.4 (4)	O2—N2—C10—C11	−61.3 (4)
N1—C7—C8—C1	−0.1 (3)	O1—N2—C10—C11	117.6 (3)
N1—C7—C8—C9	179.3 (3)		

### Hydrogen-bond geometry (Å, º)

**Table:**

*D*—H···*A*	*D*—H	H···*A*	*D*···*A*	*D*—H···*A*
N1—H1*A*···O2^i^	0.86	2.55	3.187 (4)	132

Symmetry code: (i) −*x*+1, −*y*+1, −*z*+2.
